# From screening to resection: innovation in the treatment of anorectal high-grade squamous intraepithelial lesion in high-risk patients

**DOI:** 10.1016/j.igie.2025.08.003

**Published:** 2025-08-21

**Authors:** Sofia Bragança, Paula Borralho Nunes, Sandra Pires, Luís Carvalho Lourenço

**Affiliations:** 1 Gastroenterology Department, Unidade Local de Saúde Amadora/Sintra, Amadora, Portugal; 2 Department of Pathology, CUF Descobertas, Lisboa, Portugal; 3 Instituto de Anatomia Patológica, Faculdade de Medicina da Universidade de Lisboa, Lisboa, Portugal; 4 Gastroenterology Department, CUF Descobertas, Lisboa, Portugal; 5 Gastroenterology Department, CUF Tejo, Lisboa, Portugal

We present the case of a 58-year-old woman with a history of cervical cancer diagnosed in 2022, for which she underwent hysterectomy with bilateral salpingo-oophorectomy. The patient had a high-grade squamous intraepithelial lesion and was diagnosed with human papillomavirus 16 (genotyping). As part of colorectal cancer screening, she underwent total colonoscopy, which revealed a 10-mm nongranular flat lesion classified as Paris 0-IIa, located at the anorectal junction and extending into the distal rectum (Fig. 1). Biopsies confirmed a high-grade squamous intraepithelial lesion (HSIL). High-resolution anoscopy (HRA) was subsequently performed, identifying, in addition to the previously described suspicious lesion in the anterior right quadrant (direct view), an acetowhite lesion, suggestive of dysplasia, which was biopsied and compatible with a low-grade squamous intraepithelial lesion. Following multidisciplinary discussion, the patient was referred for endoscopic submucosal dissection (ESD) instead of surgery because of the absence of malignancy suspicion and the presumed lower risk of anal sphincter injury. Despite the challenging location, the use of a distal attachment cap provided the necessary stability to perform the procedure. After marking and submucosal injection with a solution of succinylated Gelatin-based colloid (Gelofusine, B. Braun, Melsungen, Germany), methylene blue, and adrenaline (1:100,000), ESD was initiated distally using the DualKnife J (KD-650L; Olympus, Tokyo, Japan). The resection site demonstrated no defects or bleeding spots requiring treatment and was left open (Fig. 2). No postprocedural adverse events were noted, specifically, bleeding. Histopathology confirmed HSIL with focal Ki-67 and p16 staining in two-thirds of the epithelium and margin-negative (R0) resection (Fig. 2). Follow-up HRA and colonoscopy at 6 months showed no evidence of recurrence or new lesions (Fig. 3).

**Figure 1:**
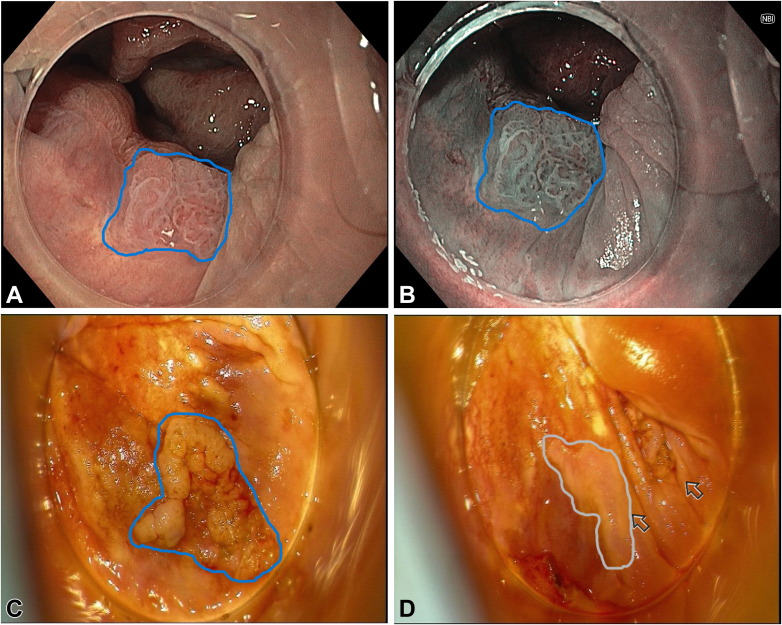
Index colonoscopy and high-resolution anoscopy findings before endoscopic submucosal dissection (ESD). **A** and **B**, Colonoscopy findings: nongranular flat lesion classified as Paris 0-IIa at the anorectal junction compatible with high-grade squamous intraepithelial lesion (HSIL) in (**A**) white-light and **B**, narrow-band imaging evaluation (*blue border around*). **C** and **D**, High-resolution anoscopy before ESD: **C**, lesion compatible with HSIL in the anterior right quadrant (*blue border around*) and **D**, acetowhite lesion compatible with low-grade squamous intraepithelial lesion (*gray border around and arrow*).

**Figure 2:**
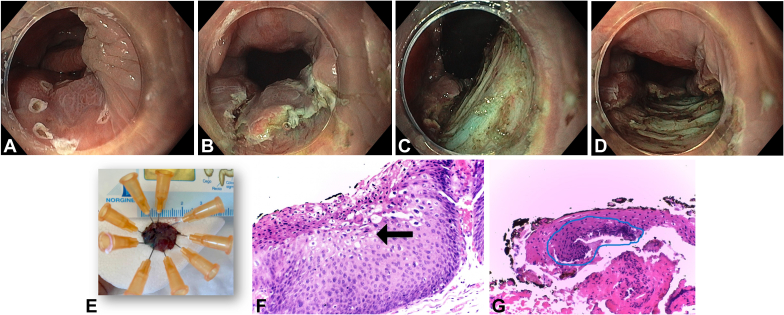
Endoscopic submucosal dissection steps and histology. **A** and **B**, Demarcation of the lesion limits and precutting, respectively. **C** and **D**, Visualization of the resection site in retroflexed view in the rectum and direct view in the anal canal, respectively. **E**, Macroscopic images of the resected specimen after en bloc resection. **F**, Histopathologic study (hematoxylin and eosin, original magnification ×200) showing the cytopathic effect of human papillomavirus (*arrow*) compatible with high-grade squamous intraepithelial lesion. **G**, Histopathologic study (hematoxylin and eosin, original magnification ×40) showing margin-negative (R0) resection (*blue border around the lesion*).

**Figure 3:**
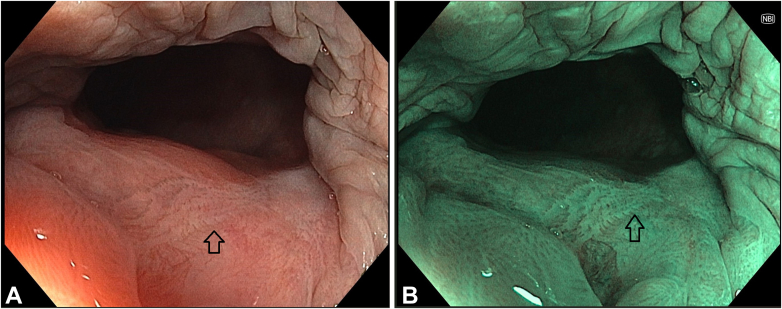
Colonoscopy at the 6-month follow-up showed the scar (*arrows*) without evidence of recurrence or new lesions on both (**A**) white-light and (**B**) narrow-band imaging evaluation.

Human papillomavirus (HPV) infection is the most common sexually transmitted infection.^1^ In certain groups, such as women with HPV-related premalignant or malignant gynecologic conditions, the infection is associated with an increased risk of adverse events in the anal canal, including HSIL and/or anal canal carcinoma.^2^ Current guidelines recommend screening for HSIL in these high-risk populations, as early detection and treatment are associated with a reduced risk of anal cancer.^2^, ^3^ Available therapeutic options for HSIL include topical treatments, ablative therapies, and surgery. However, these are associated with significant recurrence rates and, in the case of surgery, potential adverse events such as anal stenosis and incontinence.^4^ ESD is an advanced endoscopic resection technique that enables en bloc removal of lesions, achieving R0.^5^

This case highlights the adverse events of HPV infection in high-risk populations and underscores the benefit of early identification and management. We demonstrate the feasibility and potential of ESD as an innovative and promising treatment of anal HSIL, offering an alternative to conventional therapies.

## PATIENT CONSENT

The patient in this article has given written informed consent to publication of their case details.

## Disclosure

All authors disclosed no financial relationships.

## References

[1] Kombe A.J., Li B., Zahid A. (2021). Epidemiology and burden of human papillomavirus and related diseases, molecular pathogenesis, and vaccine evaluation. Front Public Health.

[2] Stier E.A., Clarke M.A., Deshmukh A.A. (2024). International Anal Neoplasia Society's consensus guidelines for anal cancer screening. Int J Cancer.

[3] Palefsky J.M., Lee J.Y., Jay N., ANCHOR Investigators Group (2022). Treatment of anal high-grade squamous intraepithelial lesions to prevent anal cancer. N Engl J Med.

[4] Richel O., de Vries H.J., van Noesel C.J.M. (2013). Comparison of imiquimod, topical fluorouracil, and electrocautery for the treatment of anal intraepithelial neoplasia in HIV-positive men who have sex with men: an open-label, randomised controlled trial. Lancet Oncol.

[5] Ng H.-I., Chen B.-H., Zhang Y.-M. (2024). Clinical application of endoscopic submucosal dissection for superficially invasive squamous cell carcinoma/high-grade squamous intraepithelial lesion involving the canal anal. Tech Coloproctol.

